# P-1659. Impact of Multidrug resistant pathogens through Pre-COVID, COVID and Post-COVID Times in a tertiary care hospital – A Retrospective cohort Study

**DOI:** 10.1093/ofid/ofaf695.1834

**Published:** 2026-01-11

**Authors:** shibi selvaraj, sanjai R, shobana selvaganesan

**Affiliations:** Jaya college of paramedical sciences, Chennai, Tamil Nadu, India; Jaya college of paramedical sciences, Chennai, Tamil Nadu, India; Jaya college of paramedical sciences, Chennai, Tamil Nadu, India

## Abstract

**Background:**

The impact of COVID-19 pandemic on extended-spectrum beta-lactamase inhibitor (ESBL) producing Enterobacteriaceae, Carbapenem-resistant organism (CRO), Methicillin-resistant Staphylococcus aureus (MRSA) and Vancomycin-resistant enterococci (VRE). This study aims to demonstrate the variations in multidrug resistance levels between the pre-COVID, COVID, and post-COVID periods.

**Methods:**

This retrospective cohort study conducted between January 2018-March 2024 at a tertiary hospital in India. January 2018-March 2020 (Pre-COVID), April 2020-July 2022 (COVID) and August 2022-March 2024 (Post-COVID). We systematically collected organism cultures, and analysed multidrug-resistant organism details using descriptive statistics.

**Results:**

During the study period, a total of 10,915 isolates were analysed, revealing an increase in multidrug-resistant pathogens during the COVID period. Specifically, the percentages of CRO were increased to 21.1%, VRE was increased to 7.2%, gram-positive was being decreased and yeast has been increased as shown in Table 1.

**Table 1:** Cumulative summary of multidrug-resistant organisms reported during Pre-COVID, COVID and Post-COVID

YEAR	TOTAL ISOLATES	GNB ISOLATES	GPC ISOLATES	YEAST ISOLATES	ESBL	CRO	MRSA	VRE
Pre-COVID (January2018-March2020)	3726	2584(69.3%)	867(23.2%)	275(7.3%)	1311(50.7%)	458(17.7%)	285(32.8%)	34(3.9%)
COVID (April2020-July2022)	4611	3308(71.7%)	995(21.5%)	308(9.3%)	1896(57.3%)	701(21.1%)	332(33.3%)	72(7.2%)
Post-COVID (August2022-March2024)	2578	1828(70.9%)	496(19.2%)	125(4.8%)	1014(55.4%)	326(17.8%)	98(19.7%)	24(4.8%)

**Conclusion:**

This study indicates a significant increase in multidrug-resistant pathogens, particularly during the COVID-19 period. The prevalence of ESBL-producing organisms, CRO, MRSA, and VRE exhibited notable spikes during this time, highlighting the impact of the pandemic on antimicrobial resistance patterns in the hospital.

**Disclosures:**

All Authors: No reported disclosures

